# Isolation of NELL 1 Aptamers for Rhabdomyosarcoma Targeting

**DOI:** 10.3390/bioengineering9040174

**Published:** 2022-04-15

**Authors:** Chengchen Duan, Helen Elizabeth Townley

**Affiliations:** 1 Nuffield Department of Women’s and Reproductive Health, Oxford University John Radcliffe Hospital, Oxford OX3 9DU, UK; chengchen.duan@wrh.ox.ac.uk; 2 Department of Engineering Science, Oxford University, Oxford OX1 3PJ, UK

**Keywords:** NELL1, aptamer, rhabdomyosarcoma, targeting antigen, metabolism suppression

## Abstract

NELL1 (Neural epidermal growth factor-like (EGFL)-like protein) is an important biomarker associated with tissue and bone development and regeneration. NELL1 upregulation has been linked with metastasis and negative prognosis in rhabdomyosarcoma (RMS). Furthermore, multiple recent studies have also shown the importance of NELL1 in inflammatory bowel disease and membranous nephropathy, amongst other diseases. In this study, several anti-NELL1 DNA aptamers were selected from a randomized ssDNA pool using a fluorescence-guided method and evaluated for their binding affinity and selectivity. Several other methods such as a metabolic assay and confocal microscopy were also applied for the evaluation of the selected aptamers. The top three candidates were evaluated further, and AptNCan3 was shown to have a binding affinity up to 959.2 nM. Selectivity was examined in the RH30 RMS cells that overexpressed NELL1. Both AptNCan2 and AptNCan3 could significantly suppress metabolic activity in RMS cells. AptNCan3 was found to locate on the cell membrane and also on intracellular vesicles, which matched the location of NELL1 shown by antibodies in previous research. These results indicate that the selected anti-NELL1 aptamer showed strong and highly specific binding to NELL1 and therefore has potential to be used for in vitro or in vivo studies and treatments.

## 1. Introduction

Neural epidermal growth factor-like (EGFL)-like protein (NELL1) has previously been shown to have an important role in enhancing or repairing tissues and bones during development and regeneration [1,2,3]. NELL1 was also found to directly or indirectly act as an important biomarker for many diseases, such as osteoporosis [4,5], metabolic diseases [6,7], neuro-related diseases including bipolar or depression [8,9], inflammatory bowel disease [10], and tumor or tumor-related membranous nephropathy [11,12,13,14]. Moreover, as a biomarker that was often seen to be upregulated with further disease progression, NELL1 shows potential beyond simply a disease indicator [13]. Previous research has shown that NELL1 was significantly upregulated in the more metastatic alveolar RMS, while only low levels were seen in the embryonal form [11]. NELL1 expression levels were also found to be directly linked to a negative prognosis [13]. NELL1 overexpression promoted tumor invasion, implying that NELL1 may function as an oncogene and play an important role in cancer progression [11,13]. Furthermore, as NELL1 was also found to act as a signaling molecule for important cell functions, previous research has shown that binding to a certain region of NELL1 could block and affect the NELL1-mediated cell adhesion [15]. Herein, we focused on the pediatric disease rhabdomyosarcoma (RMS). Current treatment of RMS relies heavily on invasive surgery, chemotherapy, and/or radiotherapy methods. These treatments do not have any targeting functionality and normally lead to off target side effects [16].

Recently, the concept of applying aptamers as a target antigen for therapeutic purposes has gained momentum [12]. Compared to antibodies that are commonly used, aptamers are much smaller and can have higher affinity and specificity and lower immunogenicity [17]. In addition, it has also been indicated that the binding of an aptamer to important cellular signaling molecules could affect the intracellular metabolism with a relatively low IC_50_ [18]. Furthermore, it is easier and cheaper to manufacture aptamers [19].

In this study, DNA aptamers that targeted NELL1 were screened and generated. NELL1 had been identified in previous microarray and microscopy studies, which suggested that it is overexpressed on the surface of target rhabdomyosarcoma cells [11]. RMS and control fibroblast cell lines were used for validation of the binding affinity and specificity of the selected anti-NELL1 aptamers (AptN). Herein, we present both multiple anti-NELL1 aptamers with good affinity and selectivity and also a robust and optimized aptamer selection protocol.

## 2. Materials and Methods

### 2.1. Cell Lines

This study tested four different cell lines, which included a normal fibroblast control cell line and three other immortalized human cancer cell lines. RH30 (American Type Tissue Culture Collection (ATCC) no. CRL-2061) was originally obtained and derived from a 17-year-old male’s bone marrow metastasis and was chosen to represent the alveolar RMS (aRMS). RD (ATCC no. CRL-7763) was originally obtained and derived from a 7-year-old female’s muscle carcinoma and was chosen to represent the embryonal RMS (eRMS). U-87MG (ATCC no. HTB-14) was derived from a human glioblastoma and chosen as a non-RMS control cancer cell line. RH30, RD, and U-87MG were all acquired from the ATCC (Manassas; VA, USA). The fibroblast cell line was kindly provided by Dr. Jo Poulton from the Nuffield Department of Women’s & Reproductive Health, University of Oxford.

### 2.2. Cell Culture

All cells were incubated in high-glucose Dulbecco’s Modified Eagle’s Medium (DMEM) (Sigma-Aldrich, Poole, UK) with 10% (*v*/*v*) Fetal Bovine Serum (FBS) (Sigma-Aldrich, Poole, UK), 2 mM L-glutamine (Gibco, Life Technologies Ltd., Renfrew, UK), penicillin (100 U/mL), and streptomycin (100 g/mL) (Gibco, Life Technologies Ltd., Renfrew, UK). T75 Tissue Culture Treated Flasks (Nunc, Thermofisher Scientific, Renfrew, UK) were used to culture the cells in a humidified 5% CO_2_ incubator at 37 °C. Cells were detached from the flask surface using 0.25% (*w*/*v*) trypsin containing 0.02% (*w*/*v*) EDTA (Sigma-Aldrich, Poole, UK).

### 2.3. Cell Survival Assay (Crystal Violet Assay)

After treatment, cells were washed with cold PBS three times before 30 min fixation in a 1% (*v*/*v*) glutaraldehyde solution. The crystal violet solution (1% (*w*/*v*) crystal violet (Sigma-Aldrich, Poole, UK) mixed in a 4:1:5 (*v*/*v*/*v*) combination of H_2_O, glacial acetic acid, and methanol) was applied to the fixed cells for 1 h at room temperature. After the staining, excess supernatant was removed. The cells were then thoroughly washed and rinsed with water until all excess crystal violet staining solution was removed. The microplate was then left to dry at room temperature for 2 days before the next step. Prior to reading, the dye in each well was resuspended vigorously with 100 µL of solubilizer (1% (*w*/*v*) SDS in 10% (*v*/*v*) glacial acetic acid). The absorbance was measured at 540 nm with a plate reader (Tecan Infinite^®^ 200 PRO, Reading, UK).

### 2.4. Cell Metabolic Assay (MTT)

Cell proliferation and metabolic activity were measured using a colorimetric MTT (3-(4,5-Dimethylthiazol-2-yl)-2,5-Diphenyltetrazolium Bromide) assay. After treatment, the medium was discarded from the microplate well. MTT medium solution (100 µL of growth medium containing 0.5 mg/mL of MTT) was then added into each well for 3 h incubation at 37 °C. Purple formazan crystals formed inside cells, and this was confirmed before the next step. All supernatant was thoroughly removed, and each well was resuspended vigorously with 100 µL of dimethyl sulfoxide (DMSO) to dissolve the formazan. Prior to reading, the microplate was incubated for 20 min and shaken for 2 min. The absorbance was then measured at 575 nm with a plate reader (Tecan Infinite^®^ 200 PRO, Reading, UK).

### 2.5. Flow Cytometry

In order to validate the location of NELL1 on the cell surface, cells were incubated with antibody and analyzed using FACS. Cells were detached from the flasks and collected by several rounds of centrifugation and PBS washing. The cells were then resuspended to a final concentration of 1 × 10^7^ cells/mL with flow cytometry staining buffer (PBS, 5% (*v*/*v*) FBS, and 0.1% (*w*/*v*) NaN_3_ (sodium azide)). The cells were subsequently incubated with 1:500 anti-NELL1 primary antibody (Abcam, Cambridge, UK) for 30 min and 1:500 Cy3-labeled anti-rabbit IgG (H + L) cross-absorbed secondary antibody (Thermofisher Scientific, Renfrew, UK) for 60 min. The excess antibody was washed away through multiple washings after every antibody incubation step.

To validate the specific binding of the anti-NELL1 aptamers with the cell surface NELL1 protein, the anti-NELL1 antibody was labeled with a fluorescent tag using Mix-n-Stain CF555 labelling kits (Biotium, CA, USA). The selected anti-NELL1 aptamers were synthesized with a 5′-biotin tag (Sigma-Aldrich, Poole, UK) and labeled with PE–streptavidin (BioLegend, CA, USA). Cells were separately incubated overnight with free PE–streptavidin, 1:200 CF555-labeled anti-NELL1 primary antibody (Abcam, Cambridge, UK), or 500 nM PE-labeled anti-NELL1 aptamers at 37 °C. The excess dye or binding ligand was washed away through multiple PBS washes after the incubation step. The whole process was conducted on ice in a dark environment. All groups of cells were fixed with 4% (*v*/*v*) PFA solution prior to testing. All cells were analyzed and compared with the FACS Calibur flow cytometer (Becton Dickinson, Wokingham, UK). The experiment was performed in replicates on three independent occasions.

### 2.6. Western Blotting

Detached cells were collected using multiple rounds of centrifugation and PBS washes. Freshly made cell lysis buffer (50 mM Tris-HCl, pH 7.4, 150 mM NaCl, and 0.01% (*v*/*v*) Triton X-100) was prepared. Directly before use, 10 μL protease inhibitor and EDTA (Life Technologies Ltd., Renfrew, UK) were added per 1 mL of lysis buffer. The cells were resuspended and lysed for 30 min on ice. The supernatant was collected with high-speed centrifugation at 4 °C and transferred into sterile 1.5 mL tubes (Eppendorf, Loughborough, UK). A Bradford Assay (Pierce™, Bio-rad, Watford, UK) was then used to determine the amount of protein in the cell lysate.

Lysates (10 µg of protein) from each cell line were separated using Novex^®^ NuPAGE^®^ SDS-PAGE Gel (Thermofisher Scientific, Renfrew, UK) in BupH Tris-HEPES-SDS Running buffer (Thermofisher Scientific, Renfrew, UK) at 180 V for 1 h. The gel transfer was performed using a Wet/Tank Blotting Transfer system (Bio-rad, Watford, UK) and a PVDF blotting membrane (Amersham GE, Buckinghamshire, UK) in ice-cold transfer buffer (200 mM Glycine, 25 mM Tris, and 20% (*v*/*v*) Methanol) at 100 V for 1 h. Ponceau S solution (0.1% (*w*/*v*) Ponceau S in 5% (*v*/*v*) acetic acid) was applied to the membrane in order to visualize and confirm successful transfer of the protein bands. The PVDF membrane was then destained using distilled water. Freshly made TBST buffer (10 mM Tris-HCl, 150 mM NaCl, and 0.05% (*v*/*v*) Tween-20) containing 5% (*w*/*v*) nonfat milk was used to block the membrane at room temperature for 2 h. The blocked membrane was then cut based on the ladder and incubated separately with 1:200 anti-Beta Actin antibody (Santa Cruz, CA, US) or 1:1000 anti-NELL1 antibody (Abcam, Cambridge, UK) in 1% (*w*/*v*) BSA in TBST overnight at 4 °C. The 1:1000 diluted goat anti-rabbit HRP-conjugated secondary antibody (Santa Cruz, CA, US) was applied for the membrane incubation after three rounds of TBST washing. Pierce ECL Western Blotting Substrate (Thermofisher Scientific, Renfrew, UK) was used to visualize the protein bands in G:Box (Syngene, Cambridge, UK).

### 2.7. Selection of Anti-NELL1 Aptamers

PEG–NHS-functionalized coverslips were prepared following the protocol from Chandradoss *et al.* with slight modifications [20]. The glass coverslips were treated with Milli-Q H_2_O, 1 M KOH, and piranha solution separately for 2 h with sonication to fully remove any possible contaminants from the surface. A mixture of APTES and methanol (1:10 *v*/*v*) was applied to allow amino bond conjugation on the coverslip surface. Biotin-PEG (2 mg) and mPEG–NHS (40 mg) (Lysanbio, AL, USA) were dissolved in 140 μL PEG buffer (100 mM of sodium bicarbonate buffer, pH 8.0) for 2 pairs of coverslips. After 3 h incubation, T50 buffer (10 mM Tris-HCl, 50 mM NaCl, and 1% (*v*/*v*) Tween-20; pH 8.0) was used to wash off excess mPEG. The functionalized coverslips were stored separately at −20 °C before use.

The NELL1 protein coating and some of the aptamer selection steps were optimized from the work of Lauridsen *et al.* [19]. NELL1 protein stock solution (25 μL and 125 μM) (R&D Systems, MN, USA) was dissolved in 30 μL PBS with 10% (*v*/*v*) glycerol. The protein solution was then applied dropwise to the PEG–NHS-functionalized coverslips on a flat, clean surface. The treated coverslips were then incubated for 40 min in a 24 °C humidifying environment before washing three times with PBST. Unreacted NHS groups were then blocked with 300 μL of 800 μM ethanolamine for 35 min in the humidifying chamber. Two more rounds of PBST washing were performed before the oligo selection step.

The 40 nucleotide random FAM-labeled oligo library (Integrated DNA Technologies, IA, USA) was synthesized following the sequencing: 5′-GGACAGGACCACACCCAGCG (40 random bases) GGCTCCTGTGTGTCGCTTTGT/36-FAM/-3′. For the negative selection of the oligo library, the treated PEG–NHS-functionalized coverslip was fully deactivated with 300 μL ethanolamine. Negative-selected nucleic acid library (100 μL) was then transferred to the NELL1 coated coverslip. The coverslip was then incubated in the clean humidifying environment overnight, washed with 800 μL PBST washing buffer, and gently dried with nitrogen gas. The selection process was monitored using a Motic 101M microscope with a Moticam 2500 camera and MHG 100B laser (Motic, Xiamen, China). The washing processes were repeated until there were only very few fluorescent dots on the whole coverslip.

The selected oligos were eluted by crushing the glass coverslip in Milli-Q H_2_O at 90 °C for 20 min. The resulting mixture was then centrifuged at 14,000 rpm to remove the supernatant that contained the oligos. An 18-cycle PCR was then performed with the Q5 HF Polymerase Mastermix (Thermofisher Scientific, Renfrew, UK) following the optimized protocol: denaturation at 98 °C for 30 s, 18 rounds of extension at 98 °C for 10 s, final extension at 72 °C for 10 min, and then held at 12 °C until the finish. The forward primer was designed as 5′-GGACAGGACCACACCCAGCG-3′, and the reverse primer was designed as 3′-ACAAAGCGACACACAGGAGCC-5′. Quality control of the PCR product was performed using 4% (*w*/*v*) agarose gel electrophoresis. The PCR product was stored at −20 °C before the next step.

The PCR products were extracted and purified with the Monarch^®^ DNA gel extraction kit (New England Biolabs, MA, USA). After quantification with NanoQuant Infinite 200 Pro (Tecan, Reading, UK), 5.33 ng of PCR fragment was used for phosphorylation and blunt-end plasmid ligation following the manufacturer’s instructions (NZYTech, Lisboa, Portugal). The ligated plasmid was quantified again before transformation. The ligated vector was then transformed into the JM109 competent *E. coli* cells following the manufacturer’s instructions (NZYTech, Lisboa, Portugal). Blue/white screening was performed using 100 μg/mL X-Gal (Promega, Wis, USA)/0.5 mM IPTG (Sigma-Aldrich, Poole, UK) agar plates containing 100 μg/mL carbenicillin. Positive colonies were picked and grown in LB media, and then the plasmids were extracted using a Monarch^®^ Plasmid Miniprep Kit (New England Biolabs, MA, USA). An extra round of validation of ligation product using PCR was performed with the same forward and reverse primers under the same conditions. After gel electrophoresis, only those ligated plasmids with amplification bands at the right size were selected. The selected plasmids were quantified, and the transformed cells were stored in 25% (*v*/*v*) glycerol in a −80°C freezer.

After the quantification, 10 μL of purified plasmids, which contained more than 200 ng of DNA, were transferred to a separate microcentrifuge tube. A T7 promoter sequencing primer (5′-(TAATACGACTCACTATAGGG)-3′) was used for sequencing. The product was then sent for DNA Sanger Sequencing (Eurofins Genomics, Wolverhampton, UK). The sequencing results were analyzed using the Addgene software (Addgene, MA, USA). The potential secondary structure and binding affinity were predicted with the DNA folding platform from the mfold web server [21].

### 2.8. Surface Plasmon Resonance Biacore Analysis

Aptamer candidates were selected from the sequencing results based on the predicted potential binding affinity. These sequences were sent off for resynthesis with 5′ biotin tags (Sigma-Aldrich, Poole, UK). Surface Plasmon Resonance Biacore T200 (GE healthcare, IL, USA) was used to quantify the selected aptamers at 37 °C. PBS was used as the running buffer to simulate an in vivo environment. A streptavidin-immobilized sensor chip Series S CAP (GE healthcare, IL, USA) was used for the quantification. The 5′-biotinylated anti-NELL1 aptamer candidates were immobilized on the chip surface following the manufacturer’s instructions at a flow rate of 100 µL/min after the chip surface regeneration. Different concentrations of NELL1 recombinant protein solution (30 nM, 300 nM, 750 nM, and 1500 nM) were injected and passed through the chip at a flow rate of 30 µL/min for 60 s. The binding affinity (Kd values) was calculated through the nonlinear fitting model using the Biacore Evaluation software and GraphPad Prism Pro fitting function.

### 2.9. Confocal Imaging of Anti-NELL1 Aptamers Specific Binding

Cells were seeded into glass-bottom imaging Petri dishes (ibidi GmbH, Martinsried, Germany) at a density of 1 × 10^5^ cells per well in DMEM growth media and left 24 h in a tissue culture incubator to allow the cells to adhere to the surface. Cells were separately incubated overnight with free PE–streptavidin (RH30 only), 1:200 diluted CF555-labeled anti-NELL1 primary antibody (RH30 only) (Abcam, Cambridge, UK), or 500 nM PE-labeled anti-NELL1 aptamers (all cell lines) at 37 °C. After incubation, the cells were washed three times with cold PBS. The whole process was conducted in a dark environment. The cells were then kept in warm PBS and stained with 0.1 µg/mL DAPI (Thermofisher Scientific, Renfrew, UK) and 1:200 diluted CellBrite™ Green Cytoplasmic Membrane Dye (Biotium, CA, USA) for 15 min separately before imaging. The cells were then thoroughly washed three times with cold PBS before fixation with 4% (*v*/*v*) paraformaldehyde solution (Bio-rad, Watford, UK) prior to imaging. The imaging was conducted with a Leica SP8 confocal microscope (Leica Biosystems, Nussloch, Germany).

### 2.10. Statistical Analysis

A statistical analysis of the experimental data was performed using Microsoft™ Excel (Microsoft, NM, USA). GraphPad Prism 8.0.2 (GraphPad Prism, La Jolla, CA, USA) was applied for graph plotting and nonlinear stimulation. All results are presented as mean ± SD. When the *p* values were less than 0.05, the difference was considered significant and shown. When two or more groups were compared, the two-tailed homoscedastic Student’s *t*-test and one-way ANOVA were applied for statistical analysis.

### 2.11. Other Software

Flow cytometry data were analyzed and compared using FlowJo VX (FlowJo, LLC, Ashland, OR, USA). The Leica Application Suite X (LAS X) was used for all relevant analysis and measurement of confocal images. ImageJ 1.53 (National Institutes of Health (NIH, Bethesda, MD, USA) was used to quantify Western blots, which were then standardized against the data from the control groups for plotting. The illustration for the Biacore chip principle was drawn with the BioRender platform (BioRender, Toronto, Canada).

### 2.12. Experimental Methodology

The researcher did not randomize or blind any experimental groups during this research. As a negative control, all samples were compared to cells cultured with the appropriate vehicle solvent.

## 3. Results

### 3.1. Cell Surface NELL1 Validation and Comparison of Expression

**Figure 1:**
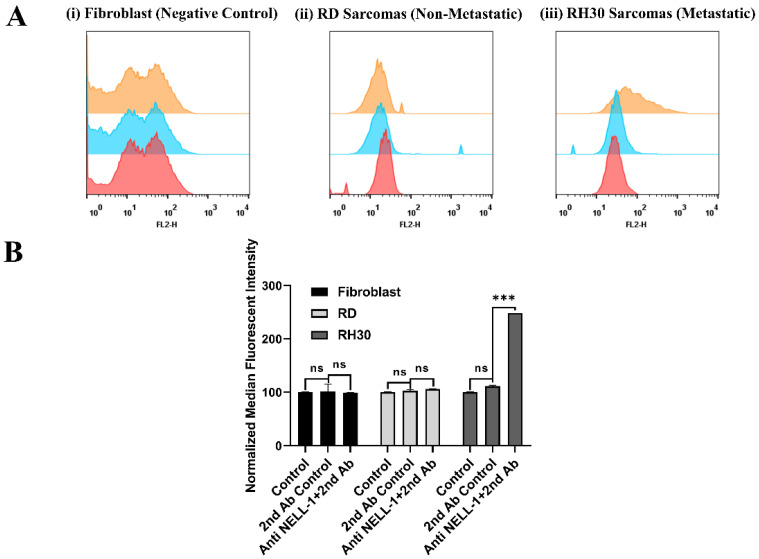
Validation of NELL1 on cell surface measured by flow cytometry. (**A**) Representative flow cytometry histogram result for fibroblast, RD, and RH30 sarcoma cells. Cells were incubated with either PBS (red), Cy3-labeled anti-rabbit secondary antibody only (blue), or anti-NELL1 primary antibody and Cy3-labeled anti-rabbit secondary antibody (orange). (**B**) Results from the normalized median fluorescence intensity of flow cytometry for fibroblast, RD, and RH30 sarcoma cells. Cells were incubated with different incubation in the PBS (control), Cy3-labeled anti-rabbit secondary antibody only (2nd Ab control), or anti-NELL1 primary antibody and Cy3-labelled anti-rabbit secondary antibody (Anti NELL1 and 2nd Ab). Data are presented as mean ± SD for each individual cell line (*n* = 3 independent experiments of triplicates). Significance was tested using a two tailed *t*-test compared to the untreated cells for each cell line (*** *p* ≤ 0.005, ns = not significant).

While previous microarray studies suggested that NELL1 is overexpressed on the surface of aRMS cells, flow cytometry experiments were undertaken for further confirmation [11]. To validate the location of the NELL1 protein at the cellular outer membrane, cells were incubated with anti-NELL1 antibody and Cy3-conjugated secondary antibody. Cells were kept alive during the whole process so the antibodies could not penetrate cell membranes. PBS control and secondary antibody-only incubation groups were applied as a control for removing the background and nonspecific binding fluorescence. Fibroblasts were chosen as being representative of normal cells. RD and RH30 were selected as being representative of eRMS and aRMS, respectively. FACS data for fibroblast, RD, and RH30 cells with different incubations are shown (Figure 1A). RH30 cells can be seen to have a significant peak shift (orange) compared with the ‘no antibody’ (red) and ‘secondary antibody only’ (blue) control groups (Figure 1A, (iii)). Conversely, there was almost no peak shift observed in the fibroblasts (Figure 1A, (i)) and RD sarcoma cells (Figure 1A, (ii)). In order to compare the difference more clearly, the median fluorescence intensity (MFI) of all flow cytometry-measured events was applied for quantification (Figure 1B). The data show that a considerable amount of NELL1 was expressed on the metastatic RH30 cell membrane with a significant increase in signal compared with the control groups. However, there was almost no NELL1 found on the fibroblasts and RD cell membranes.

**Figure 2:**
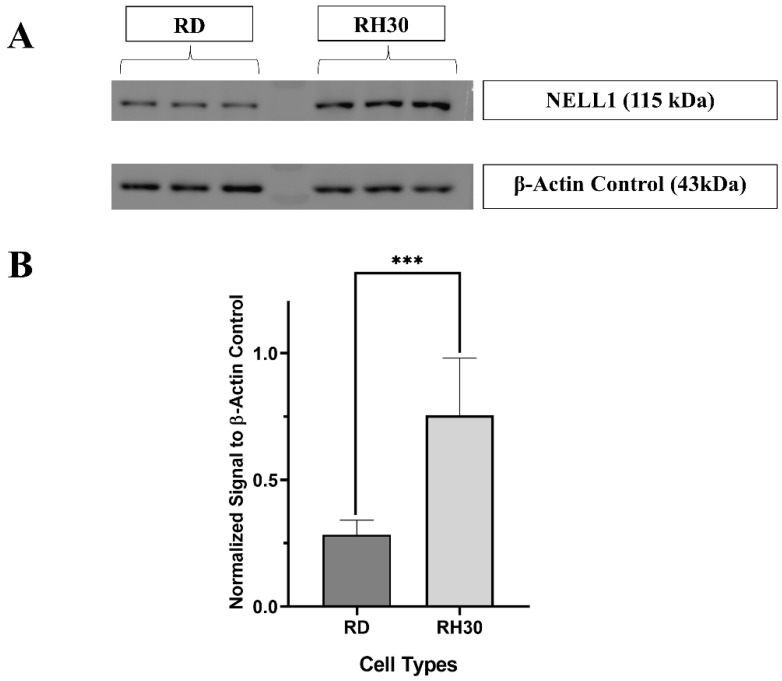
Measurement of NELL1 expression level with Western blotting. (**A**) Representative cropped Western blot comparing NELL1 expression in RD and RH30 sarcoma cells. β-Actin was applied as housekeeping gene control. The densitometry readings of each band (Tables S1 and S2) and uncropped blot (Figure S3) were included in the supplementary materials (*n* = 3) (**B**) Quantification of Western blotting showing the β-Actin normalized NELL1 expression level of different cell lines. Data are presented as mean ±SD for each individual cell line (*n* = 3). Significance was tested using a two tailed *t*-test compared to the untreated cells for each cell line (*** *p* ≤ 0.005).

As the NELL1 was shown to be localized on the outer cell membrane of RH30 (Figure 1), the expression level was then confirmed and compared by Western blotting (Figure 2). β-Actin was used as a housekeeping gene since it remains at a steady level in most kinds of mammalian cells (Figure 2A, Figure S3) [22]. Similar to the observed FACS results, RH30 sarcoma cells showed a nearly threefold higher expression level than RD cells (Figure 2B). In addition, fibroblasts were monitored as they represented normal cells in the body, which might lead to false targeting if they had a high NELL1 expression [23]. U-87MG was chosen as a further negative control as it has been shown to have very low NELL1 expression in previous studies [24]. Based on the normalized quantification blotting signal, both of the negative controls (fibroblast and U-87MG) showed a significantly lower level of NELL1 expression compared with RH30 cells (Figures S1 and S4; Tables S1 and S2).

### 3.2. Anti-NELL1 Aptamers Selection

After the NELL1 had been confirmed to have potential as the targeting protein, a FAM-labeled oligo library was used to screen for an anti-NELL1 aptamer. As the NELL1 protein was immobilized on the functionalized coverslip surface, multiple oligos remained on the coverslip after the incubation process. Several rounds of washing were conducted to remove those oligos, which had no or lower binding affinity to the NELL1 protein. Under the fluorescent microscope, the selection process could be monitored with the green fluorescence decreasing with increasing number of washing rounds. This indicated a decreasing number of the oligos remaining on the surface (Figure S2). The remaining oligos were obtained after several rounds of intensive washing. The selected oligos were extracted from the coverslip and used as the template for PCR. The right size product was then purified and ligated into blunt-end linearized plasmids and amplified in competent *E. coli* for storage and sequencing purpose.

### 3.3. Evaluation of Selected Anti-NELL1 Aptamers Candidates

**Figure 3:**
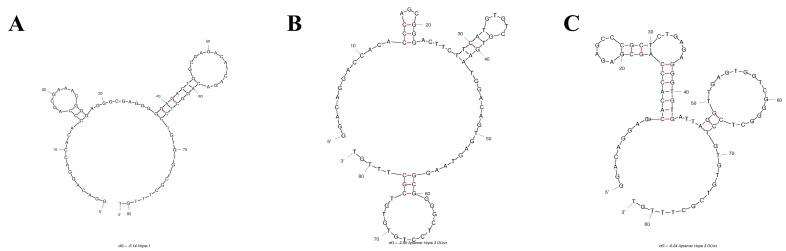
Predicted 2D Structure of anti-NELL1 Aptamer Candidates. The 2D structure of the aptamer candidates was predicted from the sequencing data and the ssDNA folding function of mFold web server. Figures show the three candidates with highest potential binding affinity: (**A**) AptNCan1, (**B**) AptNCan2, and (**C**) AptNCan3.

The secondary structure of the aptamer sequences was predicted with the mFold platform for the next step of the evaluation study [21]. Three candidates with the highest potential binding affinity, namely AptNCan1, AptNCan2, and AptNCan3, were chosen based on their functional region within the secondary structure, e.g., hairpin loops (Figure 3). At least two hairpin loops were found in the variable region of each candidate. It is noteworthy that AptNCan3 shows a multihairpin loop, which is often considered as a region likely to have a high binding affinity due to the ability to form a more complex 3D binding structure [25].

**Figure 4:**
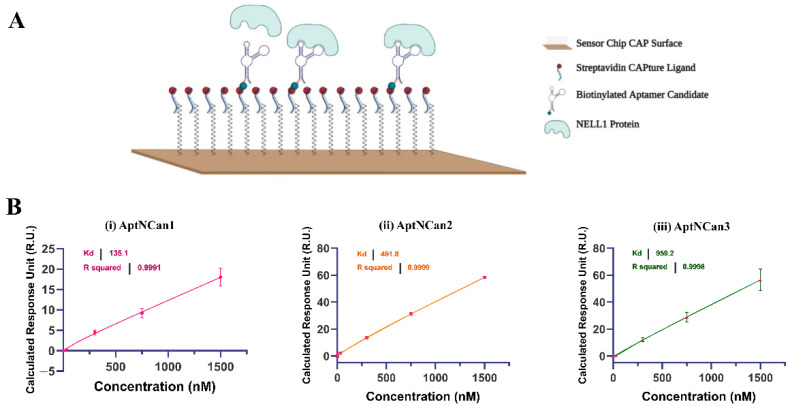
Quantification of the binding between NELL1 protein and aptamer candidates. (**A**) Illustration of the principle of using Biacore CAP microfluidic chip for the quantification of anti-NELL1 aptamer candidate to the NELL1 protein analyte. Modified from Laboratory Guideline Biacore System, 28-9615-80 AB, 11/2011, GE Healthcare Bio-Sciences AB. www.gelifesciences.com/biacore. Accessed on 10 January 2022 (**B**) Representative quantification of Kd binding affinity of different aptamer candidates fitted from the SPR binding data. Data are presented as mean ± SD for each individual candidate binding (*n* = 3).

With the help of Biacore SPR, the binding affinity of each selected aptamer candidate could be precisely quantified. Through a biotin–streptavidin linkage, the 5′-biotinylated aptamer candidates could be fixed onto the surface of Biacore CAP chips for affinity quantification with different concentrations of the recombinant NELL1 protein (Figure 4A). By deducting the baseline response unit (R.U.) value of different concentrations of NELL1 protein, the absolute R.U. values were calculated for each candidate. The Kd values that represent the binding affinity were calculated through a nonlinear fitting function. AptNCan1 demonstrated the highest binding affinity with a Kd of 135.1 nM. The other two candidates (AptNCan2 and AptNCan3) also showed good binding to the NELL1 protein, with binding limits all lower than 1 μM, i.e., a Kd of 491.8 and 959.2 nM, respectively (Figure 4B).

### 3.4. Evaluation of Selected Candidates in Vitro

**Figure 5:**
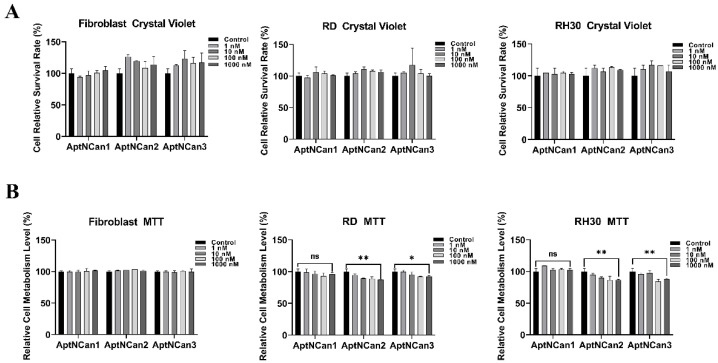
Measurement of potential effects of anti-NELL1 aptamers on cell proliferation. (**A**) Crystal violet assay results comparing the cell relative survival rate of fibroblast, RD, and RH30 sarcoma cells after treated with different concentrations of anti-NELL1 aptamer candidates for 24 h. (*n* = 6) (**B**) MTT assay results comparing the cell relative metabolism level of fibroblast, RD, and RH30 sarcoma cells after treated with different concentrations of anti-NELL1 aptamer candidates for 24 h. (*n* = 6) Data are presented as mean ±SD for each individual cell line (*n* = 3). Significance was tested using a two-tailed t-test compared to the untreated cells for each cell line (* *p* ≤ 0.05 and ** *p* ≤ 0.01, ns = not significant).

Once binding had been confirmed, further work was performed to determine the effects of the anti-NELL1 aptamers on cell proliferation and metabolism. As in the initial validation experiments, fibroblasts were chosen as being representative of normal cells. RD cells that have a very limited expression of the NELL1 protein were used as a negative control. Cells were incubated with a series of concentrations of anti-NELL1 aptamers ranging from 1 to 1000 nM for 24 h. The rates of proliferation were assessed using crystal violet staining, and metabolic activity was assessed with an MTT assay. There were no significant differences in the rates of proliferation among fibroblast, RD, or RH30 cells (Figure 5A). Metabolic activity was also not affected in fibroblast cells in the presence of the anti-NELL1 aptamers. However, the metabolic activity was reduced in both RD and RH30 cells in the presence of AptNCan2 and AptNCan3. Incubation with 1000 nM AptNCan3 showed a highly significant difference between RH30 cells (12.2% decrease in metabolic rate) and RD cells (7.7% decrease in metabolic rate) (Figure 5B).

Despite the fact that AptNCan1 showed the highest theoretical binding affinity, there was no statistically significant effect on the metabolism in either RD or RH30 cells.

**Figure 6:**
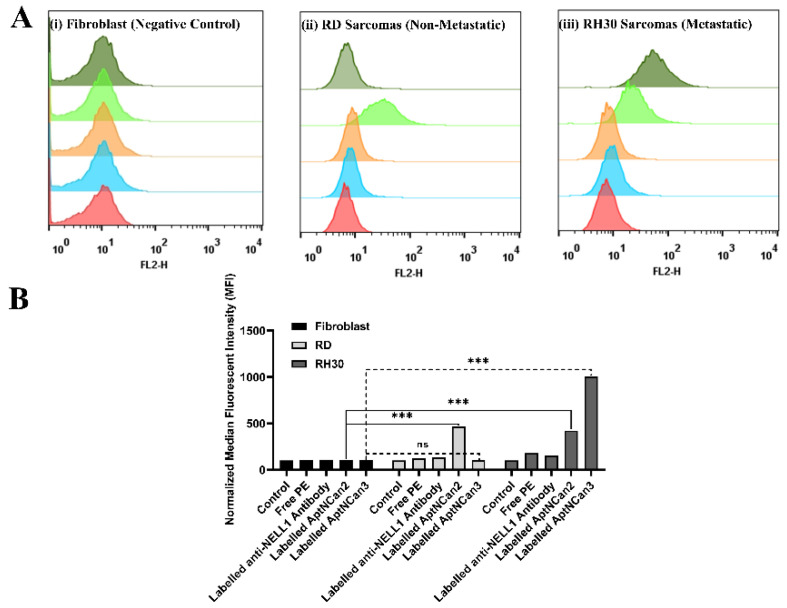
Validation of anti-NELL1 aptamer-specific binding measured by flow cytometry. (**A**) Representative flow cytometry histogram results for fibroblast, RD, and RH30 sarcoma cells. Cells were incubated with either PBS (red), free PE staining (blue), CF555-labeled anti-NELL1 antibody only (orange), PE-labeled AptNCan2 (light green), and PE-labeled AptNCan3 (dark green). (**B**) Results from the normalized median fluorescence intensity of flow cytometry results for fibroblast, RD, and RH30 sarcoma cells. Cells were incubated with different incubation in the PBS (control), free PE staining, CF555-labeled anti-NELL1 antibody, and PE-labeled anti-NELL1 aptamer 1 and 2. Data are presented as mean ±SD for each individual cell line (*n* = 3 independent experiments of triplicates). Error bars were too small to be shown. Significance was tested using a two-tailed *t*-test compared to the untreated cells for each cell line (*** *p* ≤ 0.005 and ns = not significant).

Based on the results of cell viability and the metabolism assay, AptNCan2 and AptCan3 were chosen for the next steps of specificity evaluation. Both AptNCan2 and AptNCan3 were prelabeled with 5′ biotin, and PE–streptavidin was coupled onto the 5′ ends for visualization and evaluation. To investigate the binding specificity of the NELL1 aptamers in vitro, PBS only, free PE–streptavidin, and CF555-labeled anti-NELL1 antibodies were applied as the control groups. The fibroblast negative control cell line did not show any binding with either the anti-NELL1 antibody or the aptamers AptaNCan2 or 3 (Figure 6A, (i)). RD cells showed a significant peak shift only with AptNCan2 (Figure 6A, (ii)). Both AptNCan2 and AptaNCan3 showed significant peak shifts in RH30 cells, with the latter being much greater (Figure 6A, iii)). The selectivity of both candidates for RMS cells that expressed NELL1 was proven. However, it also revealed that AptaNCan3 had significantly better specificity compared with AptNCan2 (Figure 6A).

**Figure 7:**
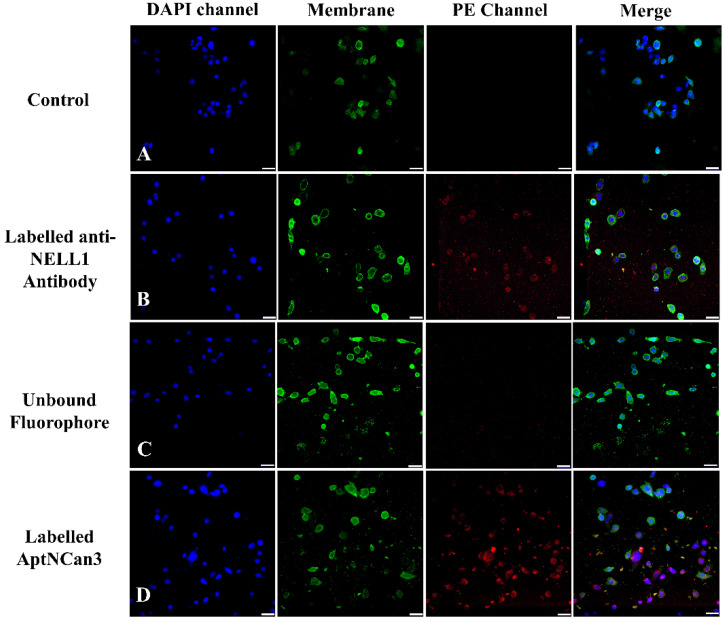
Fluorescence confocal imaging of anti-NELL1 aptamer-specific binding in RH30 sarcoma cells. The RH30 sarcoma cells were incubated overnight with (**A**) Non-labeled AptNCan3; (**B**) CF555-labeled anti-NELL1 antibody; (**C**) Unbound PE staining; and (**D**) PE-labeled AptNCan3. The cells were then thoroughly washed and fixed with 4% PFA. Scale bar, 50 μm.

The MFI data of each event (Figure 6A) were collected, and the quantitative data were illustrated in Figure 6B. All the data were normalized to the PBS control group for comparison. AptNCan2 showed significant specific binding in both RD and RH30 cells. AptNCan3 showed specific binding in RH30 cells, and the signal strength was more than twice that seen for AptNCan2. Since the number of fluorescent groups coupled to the aptamer corresponded directly to the amount of the aptamer, the MFI value was directly linked with the amount of the bound aptamer on the cell surface. Although free PE fluorophore could easily penetrate the cell membrane, it could be observed that only a very limited amount of PE signal was left after sufficient wash cycles and cell fixation (Figure 6B). This confirmed that the signal was specific to the binding of anti-NELL1 aptamers with RMS cells. Similar to the cell surface NELL1 validation data (Figure 1), the MFI of the anti-NELL1 antibody still showed a similar trend for three groups, i.e., the MFI signal was greater in RH30 compared with the fibroblast and RD groups. Although the significance among antibody-incubated groups was similar to comparable data in Figure 1, the much higher affinity of aptamer-incubated groups greatly extended the scale and minimized the apparent difference among antibody-incubated groups (Figure 6B). Based on the flow cytometry binding specificity evaluation, the AptNCan3 aptamer was used in subsequent experiments. *In vitro* localization experiments were performed using confocal microscopy after incubation of live RMS cells with the anti-NELL1 aptamer. Negative controls included unlabeled aptamer (Figure 7A) and unbound PE fluorophore (Figure 7C). A signal was only seen in the PE channel for the labeled anti-NELL 1 antibody (Figure 7B) and the labeled anti-NELL1 AptNCan3 aptamer (Figure 7D), as would be expected. Since PE–streptavidin could freely penetrate the cell membrane, all nonspecific PE signals inside the cells would be removed by washing and fixation. Cell membrane staining and DAPI cell nucleus staining were also used, and the images show that all bound signal from the PE channel was within the cytoplasm, but outside the nucleus (Figure 7). The anti-NELL1 AptNCan3 aptamer (Figure 7D) also showed a much stronger fluorescence signal compared with the antibody (Figure 7B), which also corresponded to the flow cytometry MFI data (Figure 6B).

**Figure 8:**
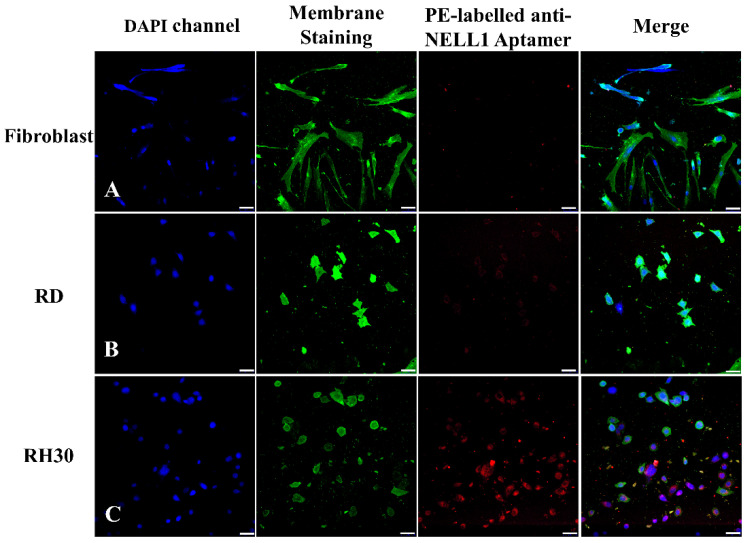
Fluorescence confocal imaging of anti-NELL1 aptamer cell-specific binding. The PE-labeled anti-NELL1 aptamer AptNCan3 was incubated overnight with (**A**) fibroblast; (**B**) RD sarcomas; and (**C**) RH30 sarcomas. The cells were then thoroughly washed and fixed with 4% PFA. Scale bar, 50 μm.

Imaging was repeated with all three cells types (Figure 8). As seen in the flow cytometry data (Figure 6), confocal imaging showed that AptNCan3 has a strong binding affinity for RH30 cells but shows almost no binding with fibroblast or RD cells (Figure 8).

**Figure 9:**
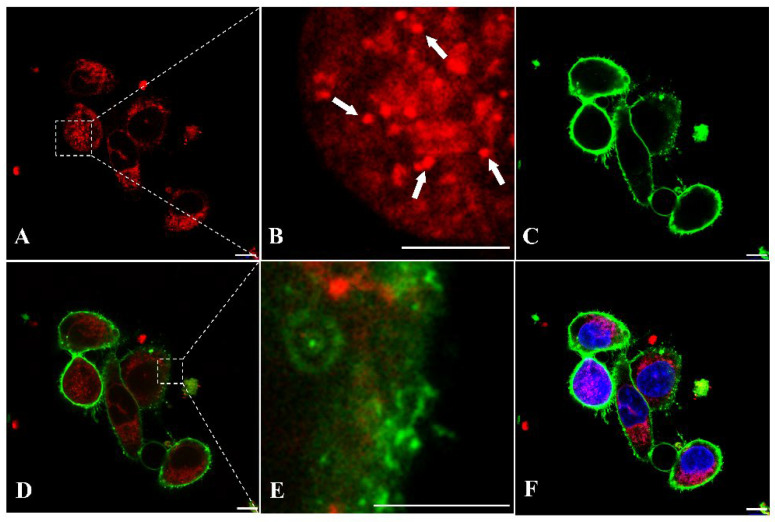
Fluorescence confocal imaging of anti-NELL1 aptamer-specific binding in RH30 sarcoma cells for binding colocalization analysis. The RH30 sarcoma cells were incubated with (**A**) PE-labeled AptNCan3 for overnight, (**B**) magnified image of the region of interest (ROI) box in A, the white arrows showing the small punctate structures, followed by (**C**) staining with CellBrite^®^ Green Membrane Staining. (**D**) merged images of A and C, (**E**) magnified image of the region of interest (ROI) box in D, and (**F**) merged images of D and the nucleus stained with DAPI. The cells were then thoroughly washed and fixed with 4% PFA before imaging. Scale bar, 10 μm.

To further investigate the cellular localization of AptNCan3, cells were imaged at a higher magnification (83×). The PE labeling of aptamers could clearly help to localize the aptamers (Figure 9A). CellBrite™ Green Cytoplasmic Membrane Staining indicated that the aptamer signal was present in the intracellular area (Figure 9C,D). Instead of evenly spreading among the cytoplasm, which most small molecules or fluorophores did, the aptamer signals showed specific overlapping with parts of the membrane signal (Figure 9E). The punctate structures indicate accumulation within a subcellular structure, e.g., lysosome (Figure 9B). Due to the multiple washes and length of incubation, most of the aptamer signals were detected inside the cells, but some were still visible on the membrane surface (Figure 9E). No NELL1 signal was observed in the nucleus or on the nuclear membrane indicating that this was not nonspecific binding (Figure 9F).

## 4. Discussion

### 4.1. NELL1 as a Biomarker for Targeting

Historically, NELL1 was recognized only as a protein with a role in osteogenic stimulation. However, more recently, NELL1 has been found to have abnormal expression levels in a number of diseases. The pediatric cancer, RMS, shows significantly higher expression levels within the metastatic alveolar RMS (aRMS) form than the embryonal form (eRMS) [11]. Moreover, it was discovered that high expression levels of NELL1 are normally linked with negative outcomes in some types of sarcomas [13]. Targeting NELL1 could therefore prove useful in the fight against cancers [12].

To be useful as a specific targeting molecule, the biomarker needs to be found on the outer surface of the cell membrane and also to be differentially expressed in cells or tissues. Although NELL1 has been identified as a secreted exocrine protein, previous studies have demonstrated that NELL1 was persistently localized in a specific way similar to a membrane protein on the outside of the cell membrane described as unconventional secretory machinery (USM). Through USM, NELL1 was normally transported and accumulated on the cell membrane before secretion. This is similar to some other secreted proteins such as fibroblast growth factor 2 (FGF2) or interleukin 1β [26,27,28]. To confirm the location of NELL1 on the outer membrane of aRMS cells, we performed flow cytometry (Figure 1). Furthermore, GFP-labeled NELL1 has previously been shown to be located on the cell outer membrane, which supported our results and provided the evidence for selecting NELL1 as a target [28].

### 4.2. Binding of NELL1 Aptamer Can Affect Cellular Metabolism

Several previous studies have directly demonstrated a relationship between NELL1 and the proliferation and metastasis of cancer cells, particularly in RMS [11,13,28]. While our study indicated that NELL1 binding by aptamers affected cell metabolism as shown by the MTT assay (Figure 5B), no similar studies have reported effects on cell metabolism, although a previous study showed successful pathway blockage that linked suppression of metabolism to the binding of an aptamer to polyphosphate kinase 2 (PPK2). The results showed that inorganic polyphosphate intracellular metabolism was significantly inhibited upon aptamer binding with an IC50 of 40 nM [18]. A scratch assay was also performed with the selected aptamer and the aptamer conjugation that showed a significant inhibitory effect by the NELL1 aptamer in the growth and mobility of RH30 cells affecting metabolism.

### 4.3. NELL1 Aptamer Specificity

The applied aptamer screening method was optimized from a protocol based on fluorescence-guided screening to immobilized NELL1 protein from a randomized library [16]. This method ensured our selected aptamers had high affinity to NELL1. However, the specific point on the protein to which the aptamer candidates bind cannot be predetermined. Our data (Figure 5B) show that both AptNCan2 and AptNCan3 could both affect the metabolism of NELL1 expressed in RMS cells, but despite having the highest binding affinity, AptNCan1 did not have any effect on cell metabolism (Figure 5B). This may be directly linked to the site of the aptamer binding on the NELL1 protein. It was also found that AptCan2 had a very similar binding for RD and RH30 cells (Figure 6), considering that both cell lines could secrete NELL1 but with a significantly different level, as shown in our study and in peer studies [11,13]. This suggested that AptNCan2 was likely to bind to another protein with a similar structure to NELL1, e.g., an isoform, given the high similarities within the NEL families. NELL2, for example, is an isoform within the NEL family, which is approximately 55% identical to NELL1. Some cell lines with relatively low NELL1 expression levels have been discovered to have a high level of NELL2 expression [3,24]. AptNCan3 was further assessed using confocal imaging, and a comparison with a commercial anti-NELL1 antibody confirmed the specificity of the aptamer to RH30 cells compared to other cell lines (Figure 6 and Figure 7).

### 4.4. The Aggregation of NELL1 in Intracellular Vesicles Was Strongly Linked to the Properties of NELL1

This study and others [3,28] showed that a large amount of NELL1 aggregated on the cell membrane, although it should be noted that NELL1 is a secreted protein rather than a membrane protein. NELL1 is normally transported to the membrane via exocytotic vesicles. This corresponds with our confocal imaging results, where the signal that corresponded to NELL1 binding ligands (red PE channel) remained within the cell membrane signal area (green), regardless of whether a NELL1-specific aptamer or the antibody was used (Figure 6, Figure 7, Figure 8 and Figure 9). Under higher magnification, it was discovered that instead of binding nonspecifically or passively diffusing into the cytosol that normally led to uniformly distribution within the cytoplasm, the ssDNA-based aptamer was specifically clustered in punctate (vesicle) structures, which was also directly associated with the properties of NELL1 and its transport mechanism (Figure 9B). The specific distribution of NELL1 protein and the specific high-affinity binding of the aptamer led to the concentration of the intracellular signal in the vesicles. This hypothesis was also supported by fluorescence and immunohistochemical results in other studies, where NELL1 showed a directional aggregation and translocation within the cytosol [3,28].

## 5. Conclusions

In conclusion, after successful validation of NELL1 expression and membrane location, several anti-NELL1 DNA aptamers were successfully screened through the optimized robust quick selection protocol. An evaluation of affinity and selectivity was performed step by step for a further selection of the screened aptamers. All three candidates were measured to have a relatively high binding affinity with a calculated Kd under 1000 nM. Two of the three candidates also showed suppression of metabolic activity in NELL1-expressing cell lines. Among those, AptNCan3 was evaluated as the best candidate that has the best balance of affinity and cell specificity. It was shown to accumulate on the membrane and in punctate structures within cells. The identification of these aptamers can permit easy, cheap, and quick manufacturing of targeting moieties toward NELL1-expressing cells, which are often those involved in cancer or other pathologies.

## Data Availability

The data presented in the study are included in the article and Supplementary Materials.

## Supplementary Materials

The following supporting information can be downloaded at: https://www.mdpi.com/article/10.3390/bioengineering9040174/s1, Figure S1: Measurement of NELL1 expression level with Western Blotting of several other cell lines, Figure S2: Representative quantified binding signal with different concentrations of NELL-1 protein with anti-NELL1 aptamer candidates, Figure S3: Full blot images of NELL1 expression level with Western Blotting of fibroblast, RD and RH30, Figure S4: Full blot images of NELL1 expression level with Western Blotting of fibroblast, U87-MG and RH30, Table S1: Measurement of densitometry readings of each β-Actin bands within Western Blotting of all tested cell lines, Table S2: Measurement of densitometry readings of each NELL1 bands within Western Blotting of all tested cell lines.
