# Supplementary material for: Isolation of NELL 1 Aptamers for Rhabdomyosarcoma Targeting
Source: Bioengineering (Basel). 2022 Apr 15;9(4):174. doi: 10.3390/bioengineering9040174 (PMC9032205; doi:10.3390/bioengineering9040174)
Supplement: Supplementary file 1 [file bioengineering-09-00174-s001.zip › bioengineering-1652403-supplementary.pdf]

Supplementary

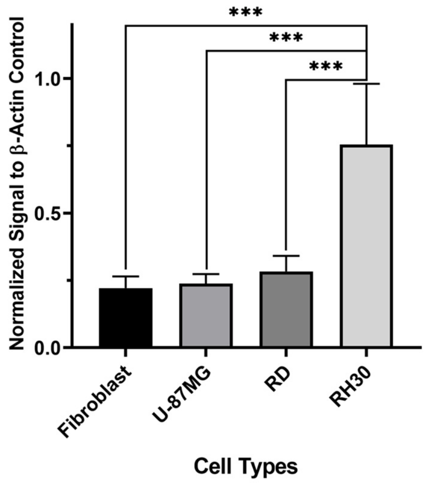

Figure S1. Measurement of NELL1 expression level with Western Blotting of several other cell lines.

Quantification of western blotting showing the β-Actin normalized NELL1 expression level of different cell lines. Data is presented at mean ±SD for each individual cell line (n = 3). Significance was tested using a two tailed t-test compared to the untreated cells for each cell line (*$p \leq 0.05$, **$p \leq 0.01$, ***$p \leq 0.005$)

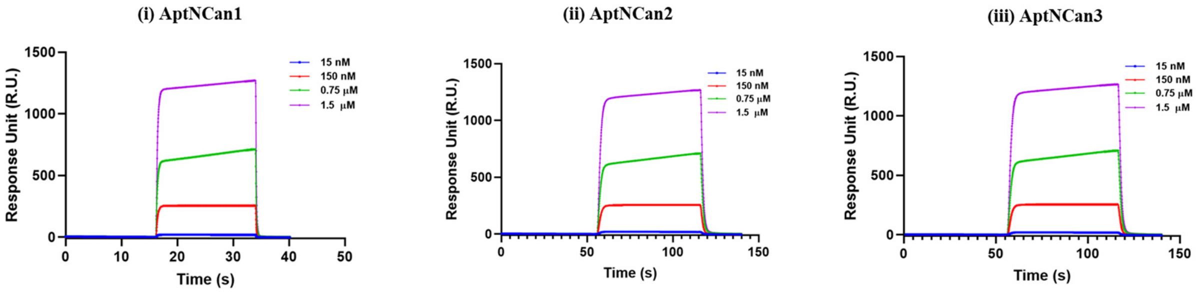

Figure S2. Representative quantified binding signal with different concentration of NELL-1 protein with anti-NELL1 aptamer candidates.

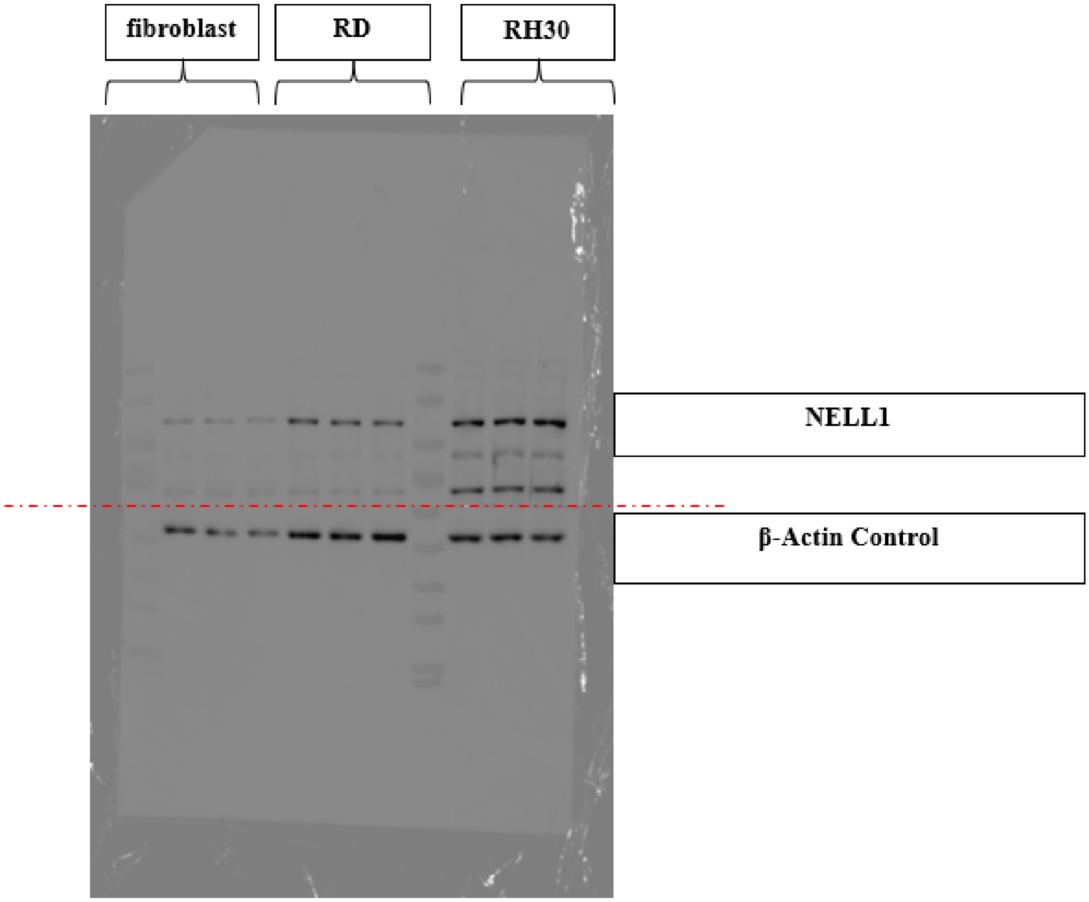

Figure S3. Full blot images of NELL1 expression level with Western Blotting of fibroblast, RD and RH30.

Western blotting showing NELL1 expression level of fibroblast, RD and RH30. β-Actin was applied as housekeeping gene control Red line showing the cutting position before the separate incubation for β-Actin and NELL1 with different secondary antibody.

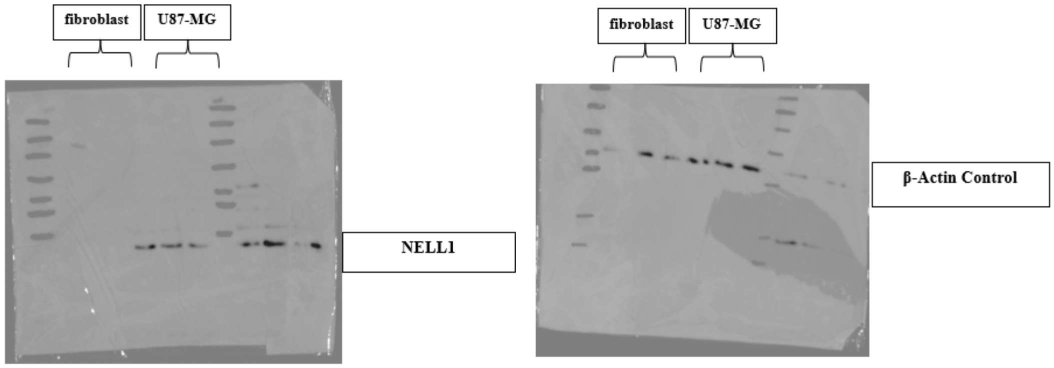

Figure S4. Full blot images of NELL1 expression level with Western Blotting of fibroblast, U87-MG and RH30.

Western blotting showing NELL1 expression level of fibroblast, RD and RH30. β-Actin was applied as housekeeping gene control The blot was cut at the last step for different incubation of secondary antibody.

Table S1. Measurement of densitometry readings of each β-Actin bands within Western Blotting of all tested cell lines.

	Fibroblast	U87-MG	RD	RH30
**Band 1**	21145.61	21629.48	33759.49	32272.44
**Band 2**	15774.97	20246.51	34191.32	32369.66
**Band 3**	14053.78	23285.39	44321.97	27870.78

Table S2. Measurement of densitometry readings of each NELL1 bands within Western Blotting of all tested cell lines.

	Fibroblast	U87-MG	RD	RH30
**Band 1**	2672.962	4205.326	11396.9	19974.44
**Band 2**	2540.79	3873.326	9929.539	20453.95
**Band 3**	2424.284	3787.912	9841.489	28318.13
